# A Rare Syndrome in Rapid Succession: A Case Report of Two Patients With Chorea‐Hyperglycemia‐Basal Ganglia (CHBG) Syndrome Within One Month

**DOI:** 10.1155/crnm/3607659

**Published:** 2026-11-10

**Authors:** Daniel R. Bashari, Aleesha Jethra, Caroline Rhame, Alexander T. Aversano

**Affiliations:** ^1^ Division of Hospital Medicine Department of Medicine Greater Baltimore Medical Center Baltimore 21204 Maryland USA gbmc.org; ^2^ Department of Medicine Greater Baltimore Medical Center Baltimore 21204 Maryland USA gbmc.org

**Keywords:** chorea, Chorea-hyperglycemia-Basal Ganglia syndrome, diabetic striatopathy, hemiballismus, hyperglycemia-induced involuntary movements, hyperglycemic chorea, hyperglycemic hemichorea, monochorea, nonketotic hyperglycemia-associated chorea

## Abstract

Chorea‐Hyperglycemia‐Basal Ganglia (CHBG) syndrome, also referred to as diabetic striatopathy, is a very rare and poorly understood cause of chorea and ballismus. Common features of CHBG syndrome include unilateral chorea or ballismus, hyperglycemia, and basal ganglia hyperintensity on neuroimaging. We present two cases of CHBG syndrome who were hospitalized at our community hospital within a 1‐month period, an occurrence that is exceedingly rare. The first case presented with subacute progressive right‐sided hemiballismus and the second case presented with acute monochorea of the left upper extremity. Both cases presented with uncontrolled Type II diabetes mellitus with HbA1c values ≥ 14%. Both cases experienced symptoms that persisted despite sleep, which is an atypical characteristic among those with CHBG syndrome. In both cases, the formal radiology reports were interpreted as normal. Retrospective review identified faint T1‐weighted hyperintensity of the left basal ganglia in Case 1. In Case 2, the consulting neurologist questioned the presence of a very subtle T1‐weighted hyperintensity of the right putamen; however, this finding was not described in the formal radiology report, could not be definitively attributed to CHBG syndrome, and its significance remains uncertain. Both cases had significant improvement in their symptoms with strict glycemic management, and both were prescribed adjunctive antichorea medications for additional symptom control. CHBG syndrome is important for physicians to know about and be able to recognize in order to arrive at a timely diagnosis and avoid unnecessary diagnostic testing.

## 1. Introduction

Chorea is a rare movement disorder characterized by involuntary nonrhythmic jerky or dance‐like movements, usually of the limbs, and sometimes involving the trunk and face. Ballismus is the more severe form of chorea, characterized by higher speed and amplitude. The basal ganglia are a group of subcortical nuclei responsible for regulating intentional motor movements. When this cluster of neurons is negatively impacted by a condition or medication (inherited or acquired etiology), it can lead to chorea or ballismus. Common causes are due to genetic conditions such as Huntington’s disease or acquired causes such as from a stroke or medications. Systemic etiologies such as genetic conditions or medications typically cause bilateral chorea, whereas focal lesions typically cause unilateral symptomatology. Hyperglycemia is a recognized metabolic cause of chorea, though it remains a rare etiology [1, 2].

We present two cases of metabolically induced chorea due to hyperglycemia, known as Chorea‐Hyperglycemia‐Basal Ganglia (CHBG) syndrome, admitted to our community hospital in early 2026 within 1‐month of each other. CHBG syndrome, also referred to as diabetic striatopathy, is a very rare cause of chorea seen in those with poorly controlled diabetes mellitus.

## 2. Case 1

A 70‐year‐old male with history of Type II diabetes mellitus, ocular acetylcholine receptor (AChR)‐positive myasthenia gravis, hypertension, and subclinical hypothyroidism presented to our emergency department (ED) with involuntary movements of his right arm and leg over the preceding one to two weeks. The patient stated that the movements were getting progressively worse over time and he had been experiencing recurrent falls. He reported that the movements persisted despite sleep. He denied any visual disturbances, headache, fever, ptosis, or respiratory symptoms. He described his symptoms being so bothersome, that he often resorted to using his left hand to control the involuntary movements of his right arm. Initial vital signs were unremarkable. Physical exam was notable for rapid large‐amplitude swinging movements of the right upper extremity, movements of the right lower extremity, and occasional muscle twitching of his right neck and face. No tremor, dystonia, or athetosis was observed. Home medications included aspirin, atorvastatin, levothyroxine, lisinopril, metformin, glimepiride, dulaglutide, and insulin glargine. The patient stated that he had only started taking the insulin weeks before presentation and was unsure of the dose. On arrival to the ED, his point‐of‐care (POC) glucose reading was 323 mg/dL. Initial laboratory results revealed a glucose value of 300 mg/dL, anion gap metabolic acidosis, and positive acetone, consistent with mild diabetic ketoacidosis. Hemoglobin A1c (HbA1c) was significantly elevated at 14.0%. Vitamin B1 level returned low, consistent with thiamine deficiency, and the patient was initiated on thiamine supplementation. Urine testing showed ketonuria and glucosuria, and the toxicology screen was negative. Please refer to Table 1 for the remainder of the pertinent clinical and laboratory findings. CTA brain/neck returned negative for any acute findings. EEG returned negative for seizure activity. The magnetic resonance imaging (MRI) brain described mild cerebral volume loss and extensive chronic microvascular ischemic changes in the supratentorial white matter, with no acute findings on the formal radiological interpretation; however, on closer inspection, faint left basal ganglia hyperintensity was noted on a single T1‐weighted image (Figure 1). A basal‐bolus insulin regimen was initiated, with subsequent improvement in the glucose levels. Neurology was consulted and did not feel that the patient’s presentation was consistent with active myasthenia and instead felt it was related to uncontrolled hyperglycemia. Neurology recommended strict glycemic control, to start a trial of low‐dose risperidone, and to follow‐up with the movement disorder neurologist as an outpatient. Symptoms improved considerably as glucose values improved, in addition to the risperidone that was added. Risperidone caused orthostatic hypotension, and thus on discharge, the patient was instead prescribed nightly olanzapine at a dose of 2.5 mg. The patient was discharged to acute neurological rehabilitation after 4 days in the hospital.

**TABLE 1:** Clinical characteristics and laboratory findings.

Clinical characteristics and laboratory findings	Reference range and units	Case 1	Case 2
Age		70	68
Sex		Male	Female
Race		Caucasian	African‐American
Duration of type II DM		7+ years	7+ years
Dominant hand		Left	Right
Presenting Symptoms		RUE chorea‐ballismus RLE chorea facial/neck twitching	LUE chorea
Symptom onset		1–2 weeks	2 days
Initial POC glucose (mg/dL)	70–120	**323**	**> 600**
Hemoglobin A1c (%)	4.8–6.0	**14.0**	**15.7**
Serum Osmolality (mOsm/kg)	277–297	286	**305**
Sodium (mEq/L)	133–145	**132**	133
Creatinine (mg/dL)	0.50–1.20	0.77	**1.29**
Glucose (mg/dL)	70–115	**300**	**644**
Serum acetone (mg/dL)	Negative	**20**	Negative
Magnesium (mg/dL)	1.59–2.56	1.91	**1.50**
Lactate (mmol/L)	0.5–1.9	1.9	**2.7**
LDL (mg/dL)	< 100	67	**174**
C‐reactive protein (mg/dL)	< 1.0	0.37	—
Vitamin B12 (pg/mL)	200–1000	365.9	—
Vitamin B1 (nmol/L)	70–180	**<** **6**	—
Copper (mcg/dL)	70–175	104	—
Arsenic, Blood (mcg/L)	< 23	< 3	—
Mercury, Blood (mcg/L)	< 4	< 4	—
Lead, venous (mcg/dL)	< 0.50	1.2	—
TSH (uIU/mL)	0.38–4.70	**9.98**	1.07
ANA Screen, IFA	Negative	Negative	—
Syphilis IgG/IgM Antibody	Nonreactive	Nonreactive	—
Lyme IgG/IgM Antibody	Nonreactive	Nonreactive	—
CMV IgG/IgM Antibody	< 0.60/< 30.00	< 0.60/< 30.00	—
ACh Receptor, Blocking (%)	< 15	**30**	—
ACh Receptor, Modulating (%)	< 32	22	—
ACh Receptor, Binding (nmol/L)	≤ 0.30	**2.87**	—

*Note:* ANA = antinuclear antibody; CMV = cytomegalovirus; ACh = acetylcholine. Bolded values indicate laboratory results outside the reference range.

Abbreviations: LDL = low‐density lipoprotein, POC = point‐of‐care, TSH = thyroid stimulating hormone.

**FIGURE 1:**
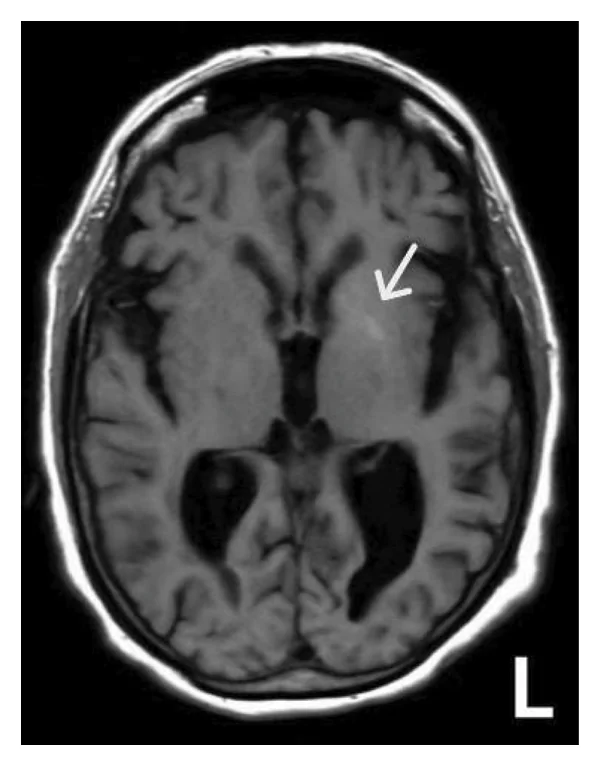
Faint hyperintensity (arrow) of the left basal ganglia demonstrated on T1‐weighted brain MRI (axial view) of Case 1.

## 3. Case 2

A 68‐year‐old female with history of type II diabetes mellitus, pulmonary embolus, morbid obesity status post sleeve gastrectomy, and endometrial cancer presented to our ED with a 2‐day history of progressively worsening left arm “shaking”. The uncontrollable movements persisted during sleep and caused frequent sleep interruption. Records showed a previously controlled HbA1c of 6.3% approximately 10 months prior; however, on presentation the patient reported noncompliance with several of her medications including her insulin, anticoagulation, and antihypertensive medications for several months. The patient did not have any past history of stroke, and the family history was negative for any neurological conditions including movement disorders. Physical examination revealed unremarkable vital signs and intermittent gross involuntary movements of her left upper extremity with preserved strength. No tremor, dystonia, or athetosis was identified on examination. Her initial POC glucose reading was above 600 mg/dL. Initial laboratory results revealed a glucose value of 644 mg/dL, HbA1c of 15.7%, lactate of 2.7 mmol/L, and a normal TSH of 1.07. Please refer to Table 1 for pertinent clinical and laboratory findings. The CT head returned negative for any acute findings. The echocardiogram was unremarkable besides stage 1 diastolic dysfunction. Carotid Doppler ultrasound returned negative for any significant carotid occlusive disease. The MRI brain described nonspecific scattered foci of T2/FLAIR signal abnormality in the periventricular and subcortical white matter of the bilateral cerebral hemispheres, felt to be compatible with chronic small vessel disease, with no acute findings such as infarction. Endocrinology and Neurology services were consulted. The patient received insulin therapy and intravenous fluids, and her glucose readings improved. It became apparent that the severity and frequency of the left upper extremity chorea improved considerably in parallel with improvement in glycemic control. Although the formal radiological interpretation of the MRI was negative for acute findings, a questionable tiny focus of T1‐weighted hyperintensity of the right putamen (Figure 2) was described by the neurologist. The patient was discharged home after 2 days in the hospital with a prescription for clonazepam to be used on an as‐needed basis.

**FIGURE 2:**
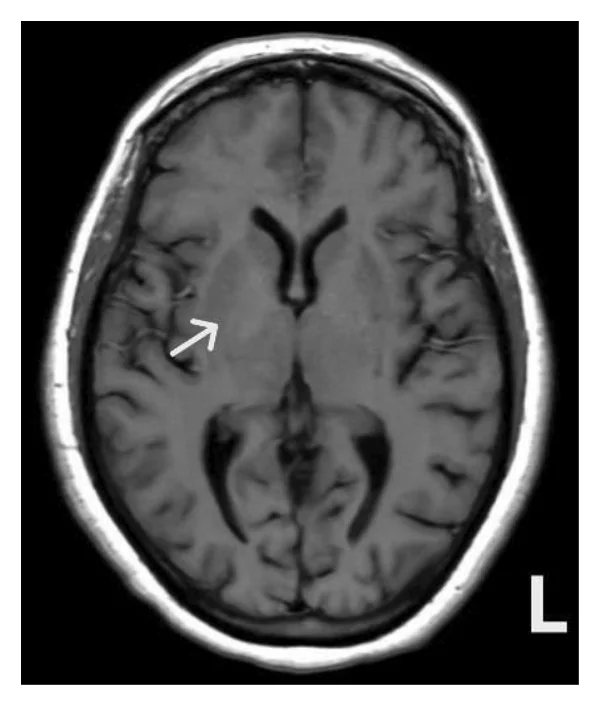
T1‐weighted brain MRI (axial view) of Case 2. The arrow indicates the region within the right putamen that was noted by the consulting neurologist as an area of questionable subtle T1 hyperintensity. This finding was not described in the formal radiology interpretation, and its significance remains uncertain.

## 4. Discussion

CHBG syndrome is a very rare manifestation of poorly controlled diabetes mellitus, a chronic disease that affects a substantial proportion of the global population. The first reported case in the literature of a patient with chorea‐ballismus associated with hyperglycemia was in 1960 [3]. The estimated prevalence of CHBG syndrome is thought to be on the order of less than 1 in 100,000 of the general population [4]. This figure is felt to be underestimated due to the rarity of the condition and its likely being underdiagnosed. To put things in perspective, a group from the Mayo Clinic found 596 cases of chorea‐ballismus from their institution over a 15‐year time span, with a total of 7 cases (or roughly 1%) of them associated with hyperglycemia [5]. In total, there have been approximately 350 cases reported worldwide as of December 2024, according to Wu and colleagues. In their article, they described approximately 300 previous cases and then presented a case series of 46 patients with hyperglycemia‐associated chorea in China over the preceding decade [6].

The exact pathophysiological mechanism for this syndrome remains unknown, although there have been proposed theories related to the hyperglycemia seen in the patient population affected. Hyperglycemia impairs cerebral autoregulation, causing hypoperfusion with activation of anaerobic metabolism and depletion of gamma‐aminobutyric acid (GABA) in the neurons of the basal ganglia. Since GABA is the main inhibitory neurotransmitter in the basal ganglia, the depletion leads to motor hyperexcitability. Hyperglycemia also causes hyperviscosity which disrupts the blood‐brain barrier, leading to transient ischemia of the basal ganglia, which is then seen on imaging as reversible radiographic findings [7, 8].

Given the literature to date, we have learned of typical characteristics of patients affected by this condition. Earlier cases described were in those of Asian descent [9–12], although more recent studies describe the syndrome in individuals with North American, Latin American, and European descent [13, 14]. Additionally, it appears that females have a slight predisposition [5–9, 13, 15]. CHBG syndrome has typically been found in patients in their sixth through eighth decades of life [13].

The initial blood glucose values in those who present with CHBG syndrome are typically elevated, although normal glucose values have also been described [7, 8]. With that said, all patients have elevated HbA1c values, indicating poor long‐term control. According to our literature review, there has been only a single case notable for a HbA1c value as low as 6.1%, though their initial POC glucose level was elevated at 315 mg/dL [15]. Most patients who present with this condition have a known diagnosis of diabetes mellitus, but some are newly diagnosed, with chorea‐ballismus being the presenting symptom. In a recent meta‐analysis of 176 patients, 30 of those patients (or 17%) were newly diagnosed with diabetes mellitus at the time they presented with CHBG syndrome [13].

In the existing literature of CHBG syndrome, most reports have described symptoms improving or resolving during sleep [6, 7, 9, 13, 16]. Conversely, both of our patients experienced symptoms that persisted and interrupted their sleep. Based on our review of the literature, only three prior case reports were identified that described patients with chorea that was not suppressed during sleep [17–19]. Persistence of symptoms during sleep therefore appears to be an infrequently reported feature of CHBG syndrome.

Neuroimaging findings include hyperintensity of the contralateral basal ganglia on computed tomography (CT) or T1‐weighted MRI, with MRI being more sensitive and specific [13]. T2‐weighted MRI images have shown variable intensity of the contralateral basal ganglia. While most patients demonstrate typical findings of basal ganglia hyperintensity, several studies have shown that a portion of patients have no evident imaging abnormalities [2, 5, 6, 11, 13, 14, 16, 18, 20, 21], which adds to the complexity of this condition. In both of our cases, the formal radiology reports were interpreted as normal. However, closer review of the imaging in Case 1 revealed faint hyperintensity of the left basal ganglia. This finding was identified retrospectively and was not reported on the initial radiology interpretation. Although the finding is consistent with imaging abnormalities previously described in CHBG syndrome, alternative radiographic explanations cannot be completely excluded, and its precise nature remains uncertain. Nevertheless, the overall presentation and outcome of the case was highly consistent with CHBG syndrome. In Case 2, the consulting neurologist questioned the presence of a very subtle area of T1 hyperintensity within the right putamen, although no corresponding abnormality was identified in the formal radiological interpretation either. Given the subtle nature of this finding and the absence of radiologic confirmation, its significance remains uncertain, and it cannot be definitively attributed to CHBG syndrome. However, despite the uncertainty surrounding the radiographic finding in Case 2, the overall constellation of findings, including the characteristic signs and symptoms, marked hyperglycemia with elevated HbA1c levels, and clinical improvement following treatment, was felt to be consistent with CHBG syndrome. Additionally, although some patients with CHBG syndrome do not display the characteristic imaging findings, this does not fully explain other discrepancies between symptoms and neuroimaging findings that may be seen, a point that has not been particularly emphasized in the existing literature. For example, cases of bilateral symptoms with unilateral imaging findings, unilateral symptoms with bilateral imaging findings [4–6, 8, 16], and unilateral symptoms with ipsilateral imaging findings [6]. Recognition of radiographically normal variants is crucial, as are cases where there are discrepancies between symptoms and radiographic findings. It has been reported that imaging abnormalities are transient and tend to correlate with improvement in the chorea‐ballismus. A delay between symptom improvement and radiographic resolution has also been described [9, 15], and interestingly, radiographic abnormalities can precede symptom onset [9, 16].

Management primarily consists of strict glycemic control, and symptoms often abate in parallel with glucose improvement. Symptom resolution occurs in most cases up to approximately 2–4 weeks after achieving euglycemia [11], although symptoms can persist in refractory cases. Some cases show resolution of symptoms with tight glycemic control only; however, some have persistent symptoms despite addressing the hyperglycemia. In these cases, adjunctive antichorea medications such as typical or atypical neuroleptics (i.e., haloperidol or risperidone) or benzodiazepines (i.e., diazepam) have been shown to be useful for control of residual symptoms [4]. The differential diagnosis of chorea is broad and includes metabolic, autoimmune, infectious, toxic, vascular, and neurodegenerative etiologies. However, both of our cases demonstrated severe uncontrolled diabetes mellitus, characteristic chorea/chorea‐ballistic movements, and clinical improvement following treatment of hyperglycemia. Given the overall clinical picture and lack of findings to suggest an alternative diagnosis, CHBG syndrome was considered the most likely etiology in both cases.

The symptoms of CHBG syndrome are not only alarming to patients and their family members but also negatively impact their quality of life. Fortunately, it is a benign disorder with a generally favorable prognosis. This syndrome is important for physicians to know about and be able to recognize in order to arrive at a timely diagnosis and avoid unnecessary diagnostic testing. Interestingly, one of our physician authors was the attending physician involved in the care of both cases. Having seen a similar case less than 1 month prior helped with the evaluation and care of the second case by arriving at a diagnosis sooner and avoiding tests that may have otherwise been ordered. Although the temporal proximity of these cases is likely coincidental and does not imply epidemiologic clustering, the occurrence of two cases of CHBG syndrome within a short period of time at a single community hospital was unusual given the rarity of the condition. Beyond providing an opportunity to compare variations in presentation and clinical course, this unexpected occurrence was emphasized in the title of our manuscript because encountering two independently presenting cases of such a rare syndrome within a 1‐month period is itself noteworthy. A limitation of this case report is the absence of long‐term clinical follow‐up and repeat neuroimaging for either case. Consequently, we were unable to assess long‐term symptom resolution or determine whether the radiographic findings evolved or resolved over time.

Despite increasing recognition, there is still much that remains unknown about this rare condition. Even with advanced imaging, biopsies, and postmortem examinations [4, 13], the exact pathophysiological mechanism is uncertain. It remains unclear why some individuals with uncontrolled diabetes mellitus develop this condition, whereas most do not. Additionally, it is not entirely clear why some lack the typical neuroimaging findings that are seen in most cases. Lastly, and perhaps the most intriguing aspect of this syndrome is that, despite being a systemic metabolic condition, it most often presents with unilateral symptoms [9, 11].

CHBG syndrome is a rare involuntary movement disorder that can be seen in those with uncontrolled diabetes mellitus. This case report adds valuable information to the existing literature on this rare condition and may contribute to a broader understanding as additional cases are reported. Further research is needed to address the gaps of knowledge surrounding this syndrome. Early recognition of this condition is imperative to help avoid unnecessary diagnostic testing and achieve prompt resolution of symptoms.

## Funding

No funding was received for this manuscript.

## Ethics Statement

This manuscript describes a retrospective report of two de‐identified clinical cases. No experimental intervention was performed. Patient information was handled in accordance with applicable institutional policies and ethical standards.

## Consent

Written informed consent for publication could not be obtained from the patients described in this report. All potentially identifying information has been removed, and every reasonable effort was made to ensure patient anonymity and protect patient privacy.

## Conflicts of Interest

The authors declare no conflicts of interest.

## Data Availability

Additional information regarding the cases described in this report may be available from the corresponding author upon reasonable request, subject to protection of patient privacy.

## References

[1] Rector W. G. , Herlong F. , and Moses H. , Nonketotic Hyperglycemia Appearing as Choreoathetosis or Ballism, Archives of Internal Medicine. (1982) 142, no. 1, 154–155, 10.1001/archinte.1982.00340140156029. 7053718

[2] Cincotta M. and Walker R. H. , One Side of the Story; Clues to Etiology in Patients With Asymmetric Chorea, Tremor Other Hyperkin Mov. (2022) 12, no. 1, 1–14, 10.5334/tohm.675. PMC8815437 35136702

[3] Bedwell S. F. , Some Observations on Hemiballismus, Neurology. (1960) 10, no. 6, 619–622, 10.1212/WNL.10.6.619. 13798206

[4] Ondo W. G. , Hyperglycemic Nonketotic States and Other Metabolic Imbalances, Handbook of Clinical Neurology. (2011) 100, 287–291, 10.1016/B978-0-444-52014-2.00021-5. 21496588

[5] Ryan C. , Ahlskog J. E. , and Savica R. , Hyperglycemic Chorea/Ballism Ascertained Over 15 Years at a Referral Medical Center, Parkinsonism & Related Disorders. (2018) 48, 97–100, 10.1016/j.parkreldis.2017.12.032. 29305082

[6] Wu X. , Fu R. , Yuan C. et al., Case Series of 46 Patients With Nonketotic Hyperglycemia-Associated Chorea: A Retrospective Follow-Up Study, Journal of Clinical Endocrinology and Metabolism. (2026) 111, no. 2, e414–e419, 10.1210/clinem/dgaf394. PMC12819872 40682440

[7] Bizet J. , Cooper C. J. , Quansah R. , Rodriguez E. , Teleb M. , and Hernandez G. T. , Chorea, Hyperglycemia, Basal Ganglia Syndrome (C-H-BG) in an Uncontrolled Diabetic Patient With Normal Glucose Levels on Presentation, American Journal of Case Reports. (2014) 15, 143–146, 10.12659/AJCR.890179. PMC3989942 24744820

[8] Dwarak S. , Prabhakar K. , Kakanale M. , Aswathanarayana A. , Sindhu B. R. , and Chorea H. , Basal Ganglia Syndrome in Diabetes–Two Rare Case Reports, Maedica. (2019) 14, no. 1, 59–62, 10.26574/maedica.2019.14.1.59. PMC6511671 31123515

[9] Oh S. H. , Lee K. Y. , Im J. H. , and Lee M. S. , Chorea Associated With Non-Ketotic Hyperglycemia and Hyperintensity Basal Ganglia Lesion on T1-Weighted Brain MRI Study: A Meta-Analysis of 53 Cases Including Four Present Cases, Journal of the Neurological Sciences. (2002) 200, no. 1-2, 57–62, 10.1016/s0022-510x(02)00133-8. 12127677

[10] Kandiah N. , Tan K. , Lim C. C. , and Venketasubramanian N. , Hyperglycemic Choreoathetosis: Role of the Putamen in Pathogenesis, Movement Disorders. (2009) 24, no. 6, 915–919, 10.1002/mds.22277. 19243026

[11] Lee S. H. , Shin J. A. , Kim J. H. et al., Chorea-Ballism Associated With Nonketotic Hyperglycaemia or Diabetic Ketoacidosis: Characteristics of 25 Patients in Korea, Diabetes Research and Clinical Practice. (2011) 93, no. 2, e80–e83, 10.1016/j.diabres.2011.05.003. 21632136

[12] Lin J. J. , Lin G. Y. , Shih C. , and Shen W. C. , Presentation of Striatal Hyperintensity on T1-Weighted MRI in Patients With Hemiballism-Hemichorea Caused by Non-Ketotic Hyperglycemia: Report of Seven New Cases and a Review of Literature, Journal of Neurology. (2001) 248, no. 9, 750–755, 10.1007/s004150170089. 11596778

[13] Chua C. B. , Sun C. K. , Hsu C. W. , Tai Y. C. , Liang C. Y. , and Tsai I. T. , Diabetic Striatopathy: Clinical Presentations, Controversy, Pathogenesis, Treatments, and Outcomes, Scientific Reports. (2020) 10, no. 1, 10.1038/s41598-020-58555-w. PMC6994507 32005905

[14] Cosentino C. , Torres L. , Nunez Y. , Suarez R. , Velez M. , and Flores M. , Hemichorea/Hemiballism Associated With Hyperglycemia: Report of 20 Cases, Tremor Other Hyperkinet Mov. (2016) 6, no. 6, 10.7916/D8DN454P. PMC4955070 27536463

[15] Lee E. J. , Choi J. Y. , Lee S. H. , Song S. Y. , and Lee Y. S. , Hemichorea-Hemiballism in Primary Diabetic Patients: MR Correlation, Journal of Computer Assisted Tomography. (2002) 26, no. 6, 905–911, 10.1097/00004728-200211000-00009. 12488734

[16] Guo Y. , Miao Y. W. , Ji X. F. , Li M. , Liu X. , and Sun X. P. , Hemichorea Associated With Nonketotic Hyperglycemia: Clinical and Neuroimaging Features in 12 Patients, European Neurology. (2014) 71, no. 5-6, 299–304, 10.1159/000357210. 24662944

[17] Pinsker J. E. , Shalileh K. , Rooks V. J. , and Pinsker R. W. , Hemichorea-Hemiballism Secondary to Non-Ketotic Hyperglycemia, Journal of Clinical Medicine Research. (2015) 7, no. 9, 729–730, 10.14740/jocmr2259w. PMC4522995 26251692

[18] Ruhangisa F. , Stephen H. , Senkondo J. et al., Acute Hemichorea in a Newly Diagnosed Type II Diabetes Patient: A Diagnostic Challenge in Resource-Limited Setting: A Case Report, BioMed Central Research Notes. (2016) 9, no. 1, 10.1186/s13104-016-2228-7. PMC4994197 27549630

[19] Park G. and Kesserwani H. N. , A Case Report of Diabetic Striatopathy: An Approach to Diagnosis Based on Clinical and Radiographical Findings, Cureus. (2022) 14, no. 5, 10.7759/cureus.25089. PMC9205274 35733455

[20] Branca D. , Gervasio O. , Le Piane E. , Russo C. , and Aguglia U. , Chorea Induced by Non-Ketotic Hyperglycemia: A Case Report, Neurological Sciences. (2005) 26, no. 4, 275–277, 10.1007/s10072-005-0471-0. 16193255

[21] Costa Hoffmeister M. , Bonavides P. S. G. , Maurer Wiercinski V. et al., Hyperglycemia-Induced Hemichorea-Hemiballismus Syndrome–A Systematic Review, Archives of Endocrinology and Metabolism. (2024) 68, 10.20945/2359-4292-2022-0413. PMC11081041 38530964

